# Case report and literature review: fetal diagnosis of vascular ring with circumflex right aortic arch and unique aortic arch branching pattern

**DOI:** 10.3389/fcvm.2025.1523356

**Published:** 2025-03-10

**Authors:** Tyler Langenfeld, Yumna Ali, Chetan Sharma, Arpit Agarwal

**Affiliations:** ^1^ Department of Pediatrics, Rainbow Babies and Children's Hospital, Cleveland, OH, United States; ^2^ Department of Medicine, University of the Incarnate Word School of Osteopathic Medicine, San Antonio, TX, United States; ^3^ Department of Pediatric Cardiology, Children's Hospital of San Antonio / Baylor College of Medicine, San Antonio, TX, United States; ^4^ Department of Pediatric Cardiology, Rainbow Babies and Children's Hospital, Cleveland, OH, United States

**Keywords:** case report, circumflex aortic arch, vascular ring, fetal echo and fetal echocardiography, cardiac CT

## Abstract

Circumflex right aortic arch is a rare aortic arch anomaly where the arch extends in a retro-esophageal pattern with a left-sided descending thoracic aorta. In the setting of circumflex right aortic arch with the ductus arteriosus connecting the left descending aorta and left pulmonary artery, a vascular ring is present and can cause compressive symptoms of the aerodigestive tract. A 33-year-old G4P3 patient underwent fetal echocardiography after obstetric ultrasound showed concern for double aortic arch. Fetal echocardiogram was suspicious for vascular ring with presumptive diagnosis of double aortic arch vs. circumflex right aortic arch. The child was born at 38 weeks gestation via induced vaginal delivery and had an uneventful postnatal course. Post-natal echocardiogram was able to diagnose vascular ring but could not fully assess the arch or branching pattern. Cardiac computed tomography angiography (CCTA) was able to definitively diagnose right aortic arch and characterize the branching pattern. To our knowledge, this is the first case reported in the literature of a circumflex right aortic arch suspected on fetal echocardiogram and postnatal echocardiogram and subsequently confirmed with CCTA. Fetal echocardiogram provides a unique opportunity to assess the aortic arch as the trachea is filled with fluid. However, circumflex aortic arch and double aortic arch can be difficult to delineate on fetal or post-natal echocardiography. CCTA is an effective modality for evaluation of the aortic arch and its branching pattern in the setting of non-diagnostic echocardiography.

## Introduction

Circumflex aortic arch is a rare form of aortic arch anomaly where a portion of the arch extends behind the esophagus while the ascending and descending aortic segments are located on the contralateral side of the spine (1). Circumflex right aortic arch refers to a retro-esophageal right aortic arch (passing over the right main bronchus to the right of the trachea and esophagus) and left-sided descending thoracic aorta. A typical vascular ring occurs in the presence of a right circumflex aortic arch with left ductus arteriosus from the descending aorta to the left pulmonary artery. This arch anomaly is extremely rare, particularly when associated with a vascular ring, which results in compressive symptoms of the aerodigestive tract during childhood (2).

In this report, we present a case of a newborn with circumflex right aortic arch first suspected on fetal echocardiogram and confirmed with post-natal imaging. Circumflex retro-esophageal aortic arch poses a diagnostic challenge and can be indistinguishable from a double aortic arch with an atretic segment on both fetal sonography and post-natal echocardiogram (3). Cardiac computed tomography angiography (CCTA) was helpful in the diagnosis as well as evaluation of the aerodigestive tract. From our extensive literature review, there are six reports on cases of a circumflex right aortic arch forming a vascular ring, none of which were diagnosed in-utero. This case is unique for both the rarity of the aortic arch branching pattern and for being the first reported case of a circumflex right aortic arch diagnosed in-utero.

## Case presentation

**Figure 1:**
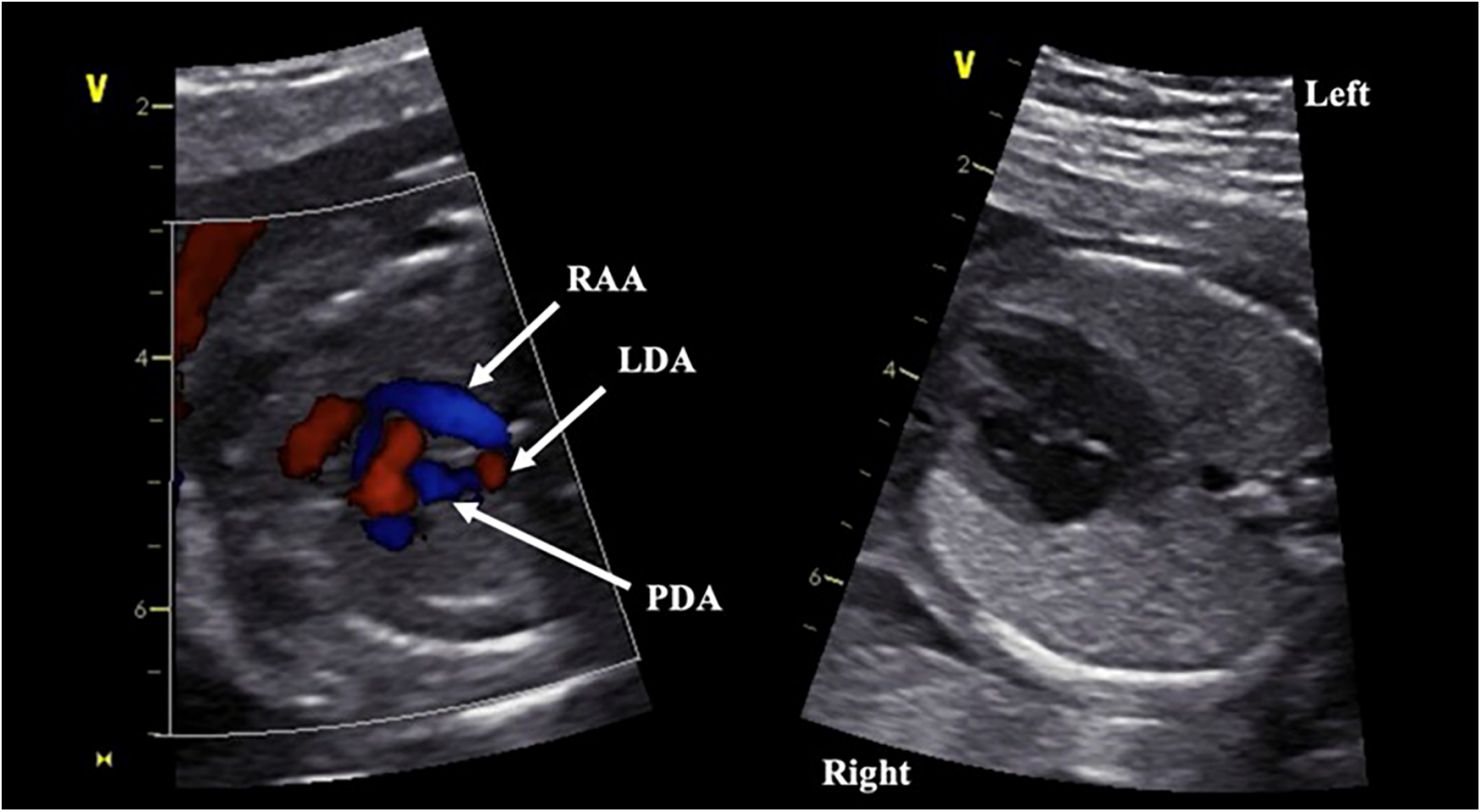
Fetal echocardiographic images at 20 weeks gestation. Panel on the left demonstrates vascular ring formed by the right circumflex aortic arch with left patent ductus arteriosus. Panel on the right demonstrates left sided descending aorta. RAA, right aortic arch; LDA, left descending aorta; PDA, patent ductus arteriosus.

A 33-year-old G4P3 patient was referred for a fetal echocardiogram at 20 weeks gestation for a concerning obstetric ultrasound indicative of a double aortic arch. Fetal echocardiogram performed by a fetal cardiologist was suspicious for a vascular ring with a presumptive diagnosis of double aortic arch vs. circumflex right aortic arch (Figure 1). The child was born at 38 weeks gestation via induced vaginal delivery and had an uneventful postnatal course with no symptoms of airway or esophageal compression.

## Imaging findings

**Figure 2:**
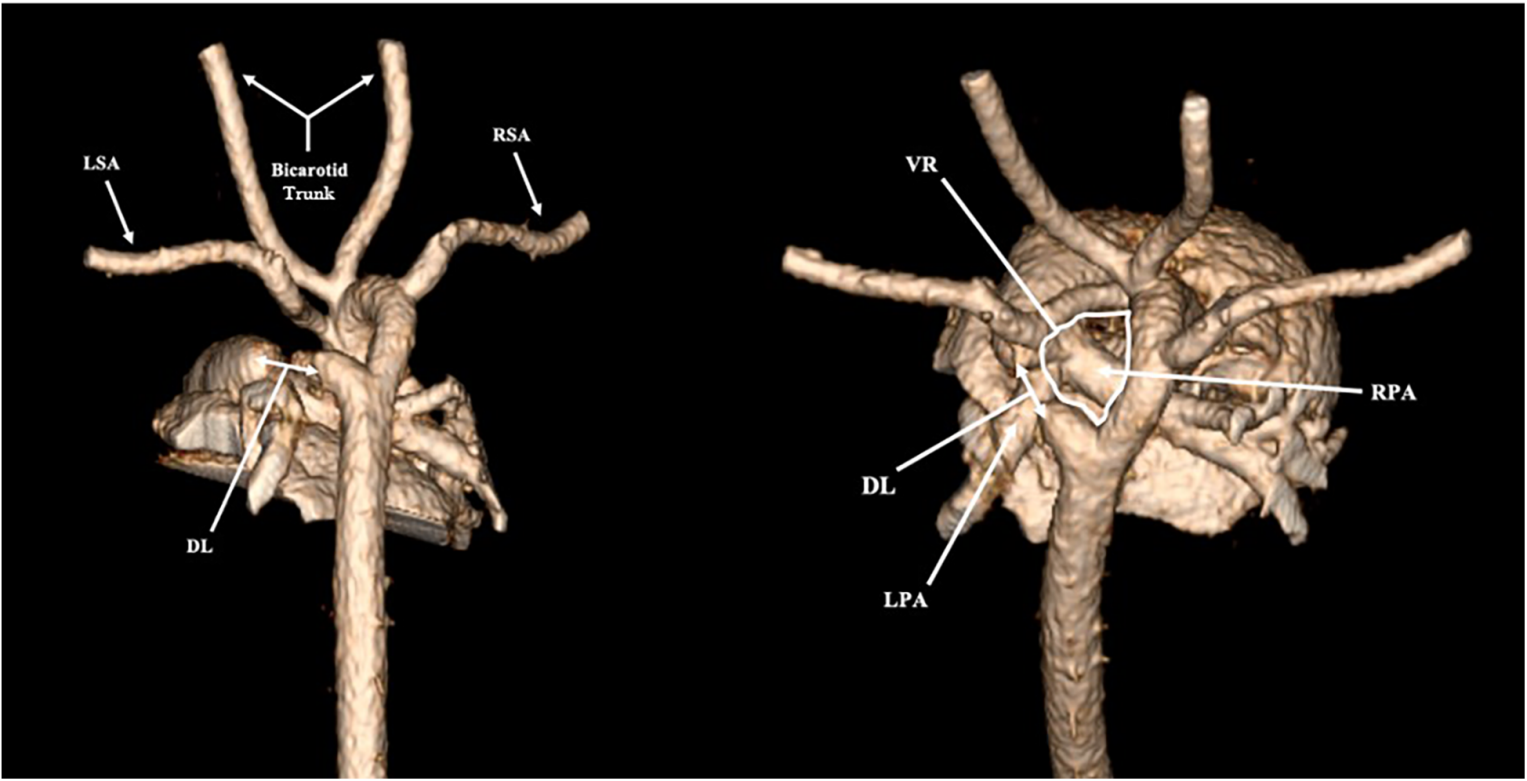
Three-dimensional reconstruction of cardiac computed tomography (CCTA). LSA, left subclavian artery; RSA, right subclavian artery; DL, ductal ligament; VR, vascular ring; LPA, left pulmonary artery; RPA, right pulmonary artery.

**Figure 3:**
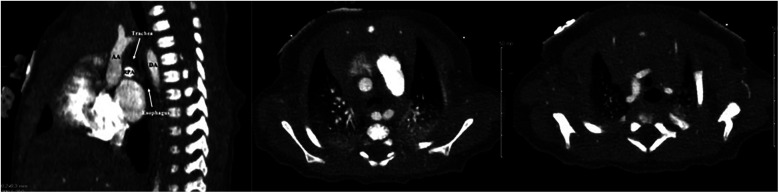
Cardiac computed tomography (CT) images demonstrating sagittal and axial views of the esophageal and tracheal compression caused by the vascular ring (middle panel taken at the level of the aortic arch for comparison to the right panel taken above the level of the aortic arch). AA, ascending aorta; DA, descending aorta; RPA, right pulmonary artery.

Fetal echocardiogram identified a right aortic arch with left-sided descending aorta, raising suspicions for a circumflex aortic arch (Figure 1). An aortic arch branching pattern was not well delineated. The differential diagnosis included right circumflex aortic arch with left ductus arteriosus vs. double aortic arch. Post-natal echocardiogram confirmed the presence of a vascular ring, but the arch behind the airway and aortic branching pattern was still not well-defined due to acoustic interface from the tracheobronchial tree and thus could not definitively diagnose the arch anomaly. CCTA was therefore performed, revealing a right aortic arch with a left descending aorta and a portion of the aortic arch extending behind the esophagus. A ductal ampulla was seen on the proximal portion of a left-sided descending aorta, which was suggestive of a right circumflex aortic arch with left ductus arteriosus (now reduced to ligamentum arteriosum). In addition, the first branch of the aortic arch was the left subclavian artery followed by a common trunk bifurcating into the left and right common carotid arteries and lastly the right subclavian artery (Figure 2). There was significant narrowing of the trachea and collapse of the esophagus at the level of the aortic arch, suggesting compression from the vascular ring (Figure 3). However, the patient was largely asymptomatic at birth without respiratory distress.

## Discussion

Several imaging modalities are available for the evaluation of vascular ring anomalies, including radiography, echocardiography, computed tomography (CT), and magnetic resonance imaging (MRI). CT has become the preferred diagnostic modality for both pre- and post-operative evaluations, as it provides excellent spatial and temporal resolution, a wide field of view, multiplanar reconstruction capabilities, and simultaneous evaluation of the airway. Echocardiography is limited in aortic arch imaging due to its two-dimensional nature, acoustic interface from the airway, and interference from bony structures such as the vertebrae (4). Additionally, circumflex retro-esophageal aortic arch can be indistinguishable from a double aortic arch with an atretic segment on both fetal sonography and post-natal echocardiography (3). However, fetal echocardiogram provides a unique opportunity to assess the aortic arch as the trachea is filled with fluid during fetal life, leading to decreased acoustic interface in comparison to a post-natal transthoracic echocardiogram.

In our case, fetal echocardiogram did demonstrate a vascular ring but could not offer a definitive diagnosis of circumflex right aortic arch. Advanced imaging was critical to identify the definitive anatomy of this rare pathology. Though tracheal and esophageal compression were present, our patient did not have any respiratory distress or feeding difficulties.

Congenital aortic arch variants and malformations result from disordered embryogenesis of the branchial arches (2). They are important to recognize as they can be associated with vascular rings, congenital heart disease, and chromosomal abnormalities (5). Vascular rings can result in compression of the tracheobronchial tree and/or esophagus due to vessels, or their atretic portions, completely encircling the trachea and esophagus (6). The vessels forming the vascular ring can include the aortic arch or arches, aortic branch vessels, pulmonary branch arteries, ductus arteriosus, or ligamentum arteriosum (5). One rare form of a vascular ring is a circumflex aorta, which occurs when the distal aortic arch courses behind the esophagus, usually at the level of T4 or T5 vertebral body, to form a descending aorta that is contralateral to the aortic arch (5–7). The aortic arch can arise on the right or left side. The vascular ring is formed from the ductus arteriosus arising from the descending aorta on the contralateral side of the arch (7, 8). In rare cases, there is a ductus arteriosus or a diverticulum of Kommerell with an aberrant subclavian artery (7, 9).

A rare type of vascular ring is formed by a circumflex retro-esophageal right aortic arch (7, 10). A circumflex right aortic arch consists of a right-sided aortic arch with a left descending aorta and left ductus arteriosus or ligamentum arteriosum (5, 11). After the distal aortic arch passes behind the esophagus, it gives rise to a left diverticulum where the left ductus arteriosus or ligamentum arteriosum arises and connects to the left pulmonary artery, forming the complete vascular ring (5). Clinical manifestations as a result of the vascular ring compressing the trachea and/or esophagus include wheezing, stridor, dysphagia, feeding difficulty, cyanosis, vomiting, and arterial insufficiency (12). Circumflex right aortic arches have been rarely reported in the pediatric population in both asymptomatic and symptomatic patients with tracheal and esophageal compression (7, 13–20). Circumflex right aortic arches have been associated with a markedly hypoplastic retro-esophageal aortic segment (7), hypoplasia and coarctation of the aorta (15, 16, 20), double aortic arch (19), ventricular septal defect with bicuspid aortic valve (15), and ventricular septal defect with coarctation of the aorta (13).

There are two typical aortic branching patterns associated with a circumflex right aortic arch, depending on the presence of an aberrant left subclavian artery (5). One pattern consists of the first aortic arch vessel as the left innominate artery followed by the right carotid artery and then the right subclavian artery (5). The other typical branching pattern consists of the following order of aortic branches from proximal to distal: left carotid artery, right carotid artery, right subclavian artery and aberrant left subclavian artery, which arises from aortic diverticulum after the aorta has crossed midline behind the esophagus (5, 7, 21). In our patient, the first branch of the aorta was the left subclavian artery, followed by a common trunk bifurcating into the left and right common carotid arteries, and lastly the right subclavian artery.

Circumflex right aortic arch with vascular ring in a symptomatic patient is managed with surgical intervention, which typically includes median sternotomy, ligation and division of the ductal ligament, and resection of the retro-esophageal portion of the aortic arch with reconstruction or anterior translocation of the aortic arch. From our extensive literature review, we were able to identify only six reports of cases of a circumflex right aortic arch forming a vascular ring, none of which were diagnosed in-utero (Table 1).

**Table 1:** Reported cases with circumflex right aortic arch.

Author/year	Age at diagnosis	Diagnosis	Clinical presentation	Management
Binsalamah et al., 2018 (19)	11 months	Double aortic arch with dominant right arch, left descending thoracic aorta (circumflex arch), tracheal compression at the level of the vascular ring, severe distal tracheobronchomalacia; right carotid and subclavian arteries rising from right arch; left carotid and subclavian arteries from left arch	Several episodes of upper respiratory tract infections, “noisy” breathing	Surgical repair via median sternotomy with aortic uncrossing
Bleakney et al., 2011 (15)	4 years	Retro-esophageal RAA, occlusion of the proximal left subclavian artery, distal aortic arch hypoplasia and coarctation	Heart murmur, blood pressure gradient between the right upper and lower extremities, dysphagia, respiratory compromise	Surgical repair via median sternotomy with resection of the circumflex arch, aortic arch advancement, and end-to-side anastomosis
Mauchley et al., 2019 (20)	Neonate	Hypoplastic retro-esophageal RAA, CoA, bicuspid aortic valve, left PDA; aortic branching pattern with the first branch being the left common carotid, followed by the right common carotid, right subclavian, and an ALSA	Multiple congenital anomalies concerning for VACTERL	Surgical repair via median sternotomy with arch reconstruction, PDA ligation and division, and vascular ring repair
Shuford et al., 1986 (11)	Case 1: 42 years	Case 1: RAA with absence of an innominate artery and separate origin of the right subclavian and right common carotid arteries, impingement of the esophagus, mild stenosis of the left subclavian artery	Case 1: Diminution of the left brachial and radial pulses as compared with the right side	None reported
	Case 2: 63 years	Case 2: Retro-esophageal RAA with left descending aorta	Case 2: Diminished pulses in the left upper extremity	
	Case 3: 60 years	Case 3: Retro-esophageal RAA with left descending aorta	Case 3: Elevated blood pressure	
Song et al., 2009 (14)	1 month	Ventricular septal defect, CoA, retro-esophageal circumflex aortic arch	Respiratory distress; grade 2 or 3 systolic murmur at the left sternal border, weak femoral pulse in comparison to radial pulse	Surgical repair via median sternotomy with anatomic reconstruction of a new left aortic arch by extended end-to-side and native tissue-to-tissue anastomosis
Subramaniam et al., 2011 (16)	4 years	Circumflex aorta with coarctation and hypoplasia of the retro-esophageal portion of the arch, Kommerell diverticulum giving rise to a stenotic left subclavian artery, vascular ring	Dyspnea on exertion, palpitations with increased precordial activity	Surgical repair via median sternotomy with anterior translocation of the hypoplastic retro-esophageal arch and creation of a left neoaortic arch

RAA, right aortic arch; CoA, coarctation of the aorta; ALSA, aberrant left subclavian artery; SVC, superior vena cava; PDA, patent ductus arteriosus.

Binsalamah and colleagues identified a double aortic arch with dominant right arch and a left descending thoracic aorta (circumflex arch) creating a vascular ring with tracheal compression (19). The patient subsequently underwent surgical repair via a median sternotomy with aortic uncrossing.

Bleakney and associates reported on a patient with a retro-esophageal right aortic arch with distal aortic arch hypoplasia and coarctation, for which the patient underwent surgical repair via a median sternotomy with resection of the circumflex arch, aortic arch advancement, and end-to-side anastomosis (15). Song and colleagues similarly performed a median sternotomy with reconstruction of a new left aortic arch via end-to-side and native tissue-to-tissue anastomosis on a patient with a retro-esophageal circumflex aortic arch (14). Additionally, Subramaniam and associates described a patient with a circumflex aorta with retro-esophageal coarctation who underwent surgical repair via median sternotomy with anterior translocation of the hypoplastic arch and creation of a left neoaortic arch (16).

Shuford and colleagues reported on three adult patients who were diagnosed with retro-esophageal right aortic arch (11). The patients were being evaluated for varying causes (chest pain, urinary retention, and elevated blood pressures) at the time of their diagnosis. None of the patients underwent surgical intervention at the time.

Following surgical intervention, patients were reported to recover without complications and post-operative imaging revealed successful repair. Of note, Mauchley and colleagues reported the development of an increasing gradient through the parachute mitral valve, but confirmed the patient continued to do well clinically (20).

## Conclusion

To our knowledge, this is the first case reported in the literature of a circumflex right aortic arch suspected on fetal echocardiogram and postnatal echocardiogram and subsequently confirmed with CCTA. This case is particularly unique for its atypical aortic branching pattern and due to circumflex right aortic arches being a rare cause of a complete vascular ring. Vascular ring due to circumflex retro-esophageal aortic arch can be difficult to distinguish from a double aortic arch with an atretic segment on both fetal sonography and post-natal echocardiography. Fetal echocardiogram provides a unique opportunity to assess the aortic arch as the trachea is filled with fluid allowing for decreased acoustic interface in comparison to a post-natal transthoracic echocardiogram. Additionally, CCTA has become the preferred diagnostic modality for both pre- and post-operative evaluations as it provides excellent spatial and temporal resolution, a wide field of view, multiplanar reconstruction capabilities, and simultaneous evaluation of the airway. Clinical presentation may vary, but this anomaly is important to recognize due to its potential to cause severe symptoms.

## Data Availability

The original contributions presented in the study are included in the article/Supplementary Material, further inquiries can be directed to the corresponding author.
